# Decay of Food DNA in the Gastrointestinal Tract: Implications for Molecular Dietary Records

**DOI:** 10.3390/nu17243865

**Published:** 2025-12-11

**Authors:** Manasvi J. Patel, Debora Regina Romualdo da Silva, Jihyun Kim, Danilo M. dos Santos, Sameer Sonkusale, Giovanni Widmer

**Affiliations:** 1 Department of Infectious Diseases & Global Health, Cummings School of Veterinary Medicine at Tufts University, North Grafton, MA 01536, USA; manasvi.patel@tufts.edu (M.J.P.); debora.silva@tufts.edu (D.R.R.d.S.); 2 School of Veterinary Medicine, São Paulo State University (UNESP), Araçatuba 01049-010, Brazil; 3 Department of Electrical and Computer Engineering, Tufts University, Medford, MA 02155, USA; jihyun.kim@tufts.edu (J.K.); danilo.dos_santos@tufts.edu (D.M.d.S.); sameer.sonkusale@tufts.edu (S.S.); 4 Sonkusale Research Labs, Tufts University, Medford, MA 02155, USA

**Keywords:** dietary recall, digestion bias, microbiota, mouse, dog

## Abstract

**Background/Objectives**: The widely recognized potential for biased responses to food frequency questionnaires and non-compliant self-reporting is motivating the search for alternative food intake records. The analysis of fecal DNA has been investigated as a potentially less biased and technically manageable method to replace or complement oral or written dietary surveys. The accuracy of fecal-DNA-based recalls critically depends on the persistence of ingested DNA of dietary origin during digestion. **Methods**: To inform the implementation of alternative molecular dietary inventories, we quantified the concentration of dietary DNA in the small intestine and in the feces of dogs, and in several sections of the mouse gastro-intestinal tract. **Results**: Using PCR assays specific for five ingredients used in commercial dog food and in mouse chow, we observed that fish DNA was most sensitive to digestion in the canine GI tract. In both species, DNA from corn and wheat was detectable in intestinal and in fecal samples. Perturbation of the mouse intestinal microbiota with antibiotics delayed the dietary DNA degradation in the GI tract. **Conclusions**: These results illustrate the limitations of DNA-based dietary recalls, underscoring their potential for generating biased information.

## 1. Introduction

It is well-known that diet plays an important role in health and disease. Collecting dietary information from nutritional study participants and subjects of epidemiological research is thus common practice. Food frequency questionnaires and dietary recalls are two leading instruments for recording food consumption. Although different in scope and application, these methods suffer from recall and reporting bias, a topic that has been extensively documented [1,2,3,4,5,6,7,8]. For instance, Klesges et al. [5] studied the bias in dietary self-reporting among different groups of subjects. This and a related publication [9] found that food intake underreporting was a function of education, gender and body mass index. Di Noia et al. [8] report the potential for social desirability to impact reporting of fruit and vegetable consumption. A comparison of two dietary recall formats found that the reported energy and nutrient intake was impacted by the survey method [10]. With the increasing the recognition of the importance of host-associated microbiota to human health, the characterization of bacterial microbiota has become an integral part of many nutritional studies. The value of fecal microbiota sequence data to human subject research is contingent on accurately documenting dietary intake because diet significantly impacts the gastro-intestinal (GI) microbiota composition [11,12,13,14,15,16]. Statistical methods for correcting systematic and random biases in assessed nutrient profiles have been investigated. Repeated dietary assessment and statistical adjustment based on validation data fall into this category [6,17,18]. Alternative and potentially less biased methods to document diet are thus desirable. Two alternative approaches which do not depend on self-reporting have been proposed; blood and urine metabolome analysis [19] and metagenomic sequencing of fecal DNA [20]. Blood and urine metabolomic data [19] have been used to obtain a poly-metabolite score which correlates with percent energy uptake from ultra-processed food. While the detection of specific metabolite markers is technically demanding, interrogating fecal DNA to obtain quantitative information on the diet consumed is technically straightforward and less costly than metabolomics. A machine learning model which uses gut microbiota profiles, together with reported dietary information, to predict the actual nutrient profile has been described [21]. This method leverages knowledge on the effect of dietary constituents on the growth of individual bacterial taxa in the GI tract.

Ecologists have long used fecal DNA to study predation and foraging habits, typically using PCR with species-specific primers [22,23,24,25,26]. To go beyond the study of predation and infer human food intake based on the abundance of fecal DNA originating from different plant and animal ingredients, it is imperative to investigate whether DNA from different food items survives digestion to a similar extent. The resistance of food DNA to chemical degradation and to enzymatic digestion is likely a function of the rate at which cellular structures and the food matrix degrade in the GI tract. The digestive degradation of DNA has been studied in various vertebrate [27,28,29] and invertebrate species [30]. In contrast, studies to assess the feasibility of using fecal DNA markers to validate or replace dietary recalls in humans are uncommon and lack information on the extent of digestion bias [22]. In vitro studies have revealed that the DNA degradation by intestinal nucleases is impacted by food composition [31], highlighting the potential for differential DNA digestion affecting the interpretation of DNA-based recalls. Intestinal DNases in the GI tract originate from secretory cells in various GI organs or from the microbiota. DNase activity in the GI lumen has been hypothesized to be important for the removal of extracellular DNA which can contribute to intestinal inflammation [32].

An interesting and potentially unbiased source of dietary information has been obtained by sequencing fecal metagenomes [20]. As expected, shotgun metagenome sequencing detected DNA from ingested food, leading the authors to suggest an entirely sequence-based approach to replace conventional recalls. To probe the concept of what is sometimes referred to as “digestion bias” and inform the development of alternative dietary surveys, we investigated the rate of decay of DNA from different food sources in the GI tract. To this aim, DNA extracted from food, from the small intestine and from feces of dogs and mice was quantified.

## 2. Materials and Methods

### 2.1. Animal Experiments

#### 2.1.1. Mice

Four B6.Cg-Il17ratm2.2Koll/J mice bred in-house were fed non-autoclaved conventional 18% protein rodent chow (Teklad, Tampa, FL, USA). They were housed in two cages. Group 1 mice (*n* = 2) received streptomycin (5 g/L), bacitracin (0.25 g/L), neomycin (1 mg/mL) and vancomycin (0.5 mg/mL) with 0.5% sucrose in drinking water for 8 days. Group 2 mice were given autoclaved water without antibiotics or sugar. The number of animals was chosen empirically. The lack of expected effect size precluded any sample size calculation. At the end of the 8-day treatment period, mice were euthanized. The intestinal tract was removed and portions of approximately 100 mg (mean = 0.099, SD = 0.013, *n* = 26) from the jejunum, cecum, colon and feces were stored at −20 °C until further processing. The experiment was approved by the Tufts University Institutional Animal Care and Use Committee under protocol G2024-52.

#### 2.1.2. Dogs

Intestinal content and fecal samples were obtained from two dogs enrolled in a clinical trial to test an ingestible sampling device conducted at the Cummings School of Veterinary Medicine. The device is similar to a conventional pill and is essentially as previously described [33] except for a modified valve. A detailed description of the pill and the analysis of the microbiota samples collected with this device are the focus of a separate publication. The number of dogs (*n* = 2) was not based on any sample size calculation but reflects the availability of samples collected for a different study. Client-owned dogs were orally given two pills each and asked to chill the feces collected over a 72 h period and return them to the clinical trial office. Dogs were routinely fed a Purina ProPlan weight management diet by their owners. According to product specifications, the main ingredients of this diet are corn, wheat, soya, fish and chicken. The trial was approved by the Tufts University Institutional Animal Care and Use Committee under protocol G2024-102.

### 2.2. Molecular Analyses

The abundance of six food ingredients in diet, intestine and feces was estimated using PCR with published primers specific for corn, wheat, soya, yeast, chicken and fish (Table 1). DNA was extracted from soya beans (*Glycine max*), wheat grains (*Triticum aestivum*), corn grains (*Zea mays*), chicken meat (*Gallus gallus*), salmon (*Salmo salar*) and rodent chow. Wheat grains, soya beans and corn grains were washed with distilled water and crushed to a powder. Approximately 100 mg of each ingredient was extracted with the QlAmp PowerFecal DNA Pro kit (Qiagen, Germantown, MD, USA) per the manufacturer’s protocol. DNA was extracted from raw chicken and fish (salmon) using the same kit. Extracted DNA was recovered in 100 µL. The PCR conditions for each ingredient are given in Table S1. Amplicons were Sanger-sequenced. Confirming the specificity of the PCR assay, the most significant BLAST (https://blast.ncbi.nlm.nih.gov/Blast.cgi, accessed on 1 October 2025) match for each assay is shown in the table. The specificity of each assay was confirmed in cross-specificity experiments (Figure S1). DNA was also extracted from Teklad 18% protein rodent chow and from the ProPlan weight management diet (Purina, St. Louis, MO, USA). DNA extract from food was used as a positive control in addition to DNA extracted from individual food items. PCR without added DNA was included as negative control in each experiment. Using the same procedure, DNA was extracted from the mouse intestinal tissue and feces samples weighing approximately 100 mg, from the dog intestinal content collected by the ingestible pills and from the feces from the same two dogs. PCR products were fractionated by electrophoresis in 2% agarose and visualized with GelRed (Biotium, Fremont, CA, USA). Gels were imaged under UV illumination. A 100 bp DNA ladder was used for size comparison. To obtain a standard curve for converting PCR Crossing Points (Ct) into DNA concentration, three 10-fold serial dilutions of the positive control DNA were prepared with DNA-free water. DNA dilutions were typically amplified in triplicate so that a standard curve is based on at least nine data points. Ct values were generated using a Quantinova SYBR Green RT PCR mastermix (Qiagen) in a Mic Real Time PCR cycler (Biomolecular Systems, Potts Point, Australia). Bacterial DNA was quantified in a similar manner using real-time PCR with published generic primers targeting the V4 variable region (forward: gtgccagcmgccgcggtaa; reverse: cacggtcgkcggcgccatt) [34], but lacking the adaptor, barcode, pad and link nucleotides. The following PCR conditions were used to amplify the V4 rRNA gene region: 1 m at 95 °C followed by 33 cycles of 5 s at 95 °C, 30 s at 55 °C, 20 s at 72 °C and a final 5 m extension at 72 °C. V4 qPCR standard curves were obtained by amplifying three 10-fold serial dilutions of DNA from a calibrated synthetic bacterial population (BEI Resources, Manassas, VA, USA, cat no. HM-782D).

### 2.3. Statistical Analysis

A paired *t*-test was used to test the effect of antibiotics on the concentration of bacterial DNA in the mouse intestine.

## 3. Results

**Figure 1:**
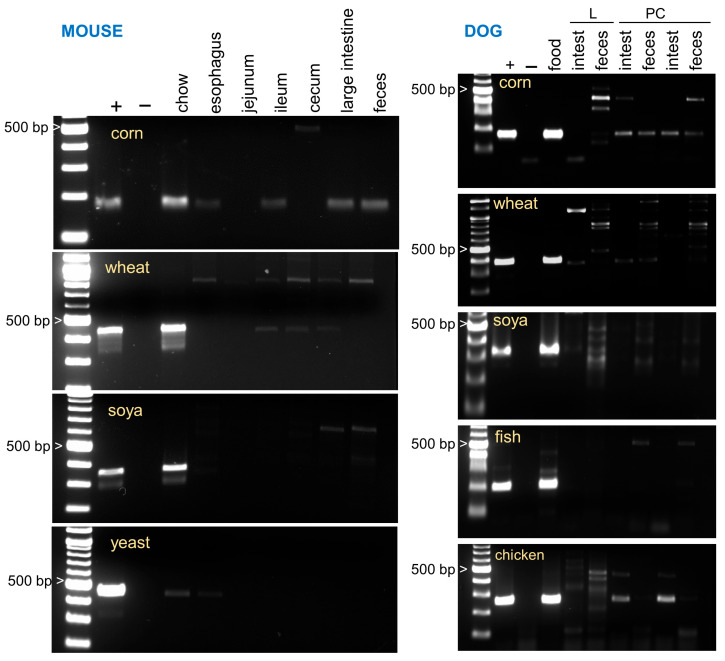
Rapid decay of food DNA in the murine and canine GI tract. **Left**: Mice fed the Teklad 18% Protein Rodent Diet (cat. # 2018SC) were sacrificed and the GI tract was immediately recovered. DNA extracted from different GI segments of approximately equal weight was subjected to PCR analysis. Primers specific for three main chow ingredients and for yeast were used. **Right**: Two healthy dogs (L and PC) enrolled in a clinical trial to test the function of autonomous sampling devices [33] were each given two pills by mouth. Content from the small intestine sampled by these devices was recovered and analyzed by PCR using the food-specific primers shown in Table 1. The lane labeled “food” shows the amplicons amplified from DNA extracted from the Purina ProPlan weight management diet. Leftmost lane, 100 bp DNA ladder; +, positive control, amplicon obtained from each ingredient; − negative PCR controls, no DNA added.

**Figure 2:**
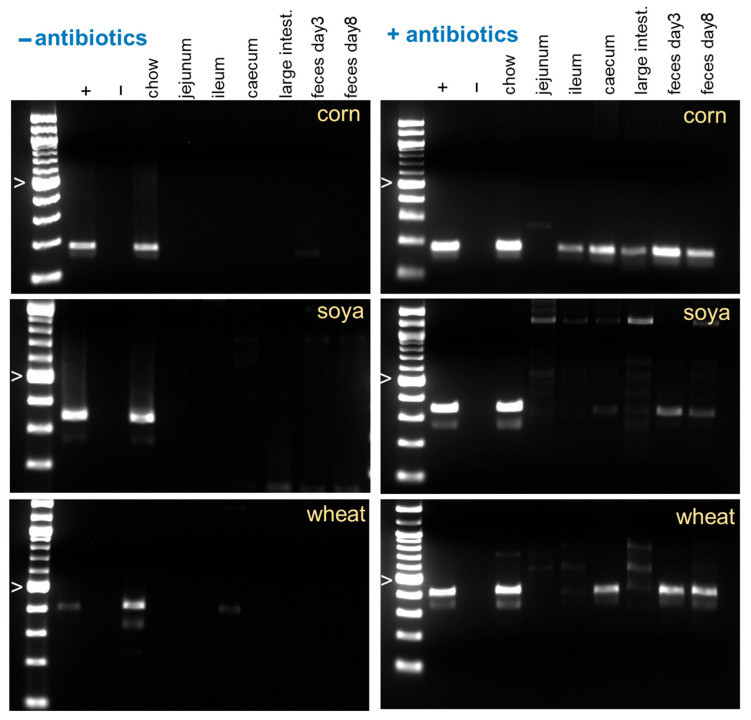
The intestinal microbiota accelerates food DNA decay. Mice fed the Teklad 18% Protein Rodent Diet were orally treated for 8 days with streptomycin, bacitracin, neomycin and vancomycin in the drinking water. This treatment was intended to deplete the bacterial microbiota [40]. Mice were sacrificed after 8 days of treatment and DNA was extracted from four GI organs of approximately equal weight. The abundance of DNA from three ingredients was analyzed by PCR (Table 1 and Table S1). Compared to control mice (**left**), DNA decay is visibly impaired in antibiotic-treated mice (**right**). Symbol “>” indicates 500 bp marker. + and − indicate positive and negative PCR controls.

**Figure 3:**
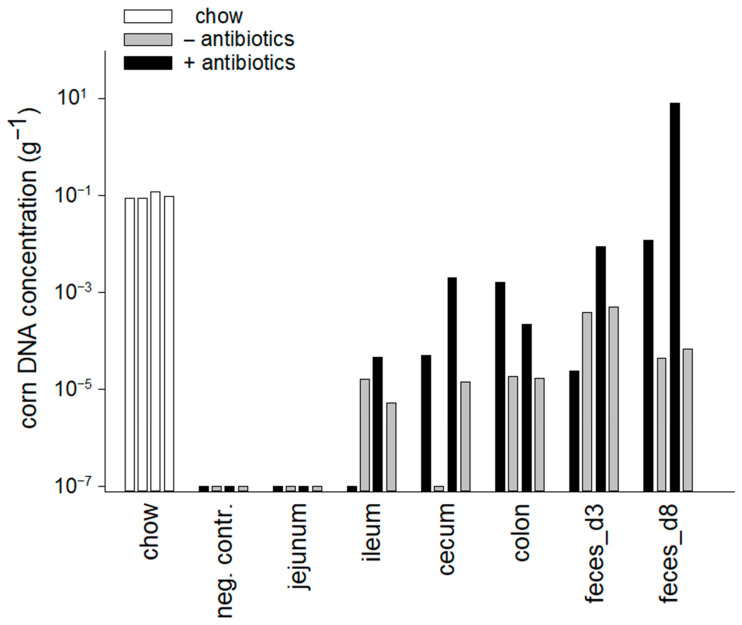
Persistence of corn DNA in antibiotic treated and untreated mice. Consistent with the gel images (Figure 2), corn DNA concentration in antibiotic-treated mice exceeds that observed in untreated controls. qPCRs were duplicated as indicated with bars of matching shading. Four estimates of corn DNA concentrations in the chow are indicated with empty bars. Each bar represents the result of one independent PCR. Feces were collected after 3 and 8 days of antibiotic treatment.

We analyzed food DNA degradation in two animal species, mouse and dog. As apparent in Figure 1, in the mouse, chow DNA is rapidly digested with the exception of corn DNA which persists at a detectable concentration in most samples. DNA extracted from unprocessed ingredients (corn, wheat, soya, yeast) and from rodent chow was consistently amplified with each of the four primer pairs. To assess the role of the murine intestinal microbiota in food DNA digestion, two additional experiments were performed with mice pre-treated for eight days with antibiotics to deplete the microbiota. This experiment is relevant to begin understanding the extent to which digestion bias is affected by intestinal dysbiosis. Figure 2 and Figure S2 illustrate the effect of the treatment on digestion of dietary DNA. Consistent with what is known about the ability of intestinal anaerobic bacteria to digest plant polysaccharides and the resulting exposure of food DNA to luminal nucleases, in antibiotic-treated mice the decay of DNA was clearly reduced. To ensure the accuracy of the information obtained from the gels, the corn DNA concentration in the GI tract of these mice was quantified using qPCR (Figure 3). The results of this analysis broadly agree with the gel images in showing the persistence of corn DNA in the mouse GI tract and a trend towards a higher target concentration in antibiotic-treated mice.

In addition to the analysis of DNA originating from food ingredients, we also quantified the bacterial DNA concentration using generic PCR primers flanking the V4 region of the prokaryotic 16S rRNA gene. This analysis (Figure S3) shows that the treatment depressed the bacterial DNA concentration in the proximal GI tract, but not in the cecum and in the feces. The antibiotic effect is, however, statistically not significant (paired T, *p* = 0.7, *n* = 5). A second independent comparison showed a similar pattern. The ratio of 16S DNA concentration in treated vs. untreated mice was 4 × 10^−2^, 1 × 10^−3^, 6.8, 1.1 and 1.0 in the jejunum, ileum, cecum, colon and feces, respectively, again showing a lack of antibiotic effect in the distal GI tract. We conclude from the results obtained with V4 qPCR that bacterial DNA increased from proximal-to-distal locations and that the antibiotics do not deplete bacteria, particularly in the distal portions of the GI tract.

**Figure 4:**
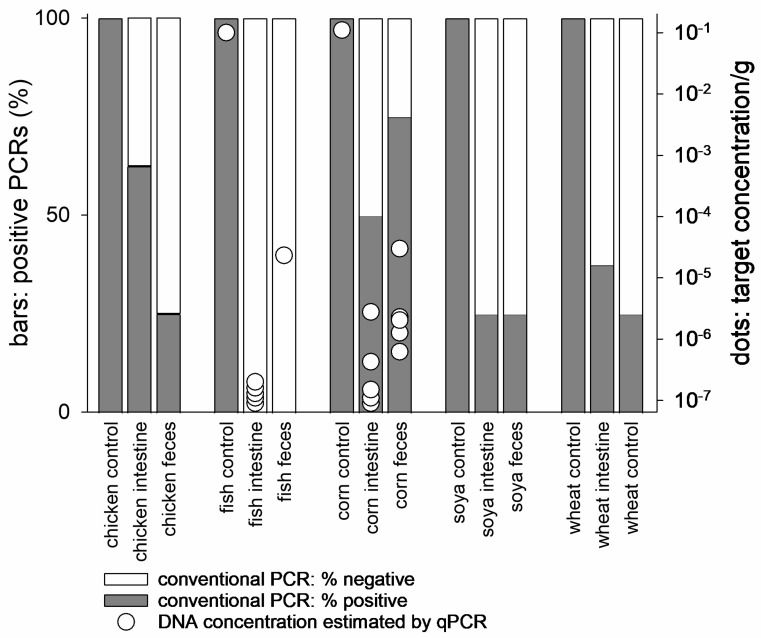
Cumulative categorical and quantitative results of ingredient-specific conventional PCR and qPCR analysis of canine intestinal and fecal samples and DNA from five food ingredients. Amplicons were fractionated on agarose as shown in Figure 1 and visually scored as positive or negative as indicated with white and shaded stacks. Intestinal and matching fecal samples were collected from two dogs. The analysis is based on 95 PCRs, 40 from intestinal samples, 40 from feces and 15 from dog food. A total of 19 PCRs were run for each of the five ingredients. For each ingredient, the left bar shows the proportion of positive PCRs obtained with food DNA. The middle and right bars show the results obtained with intestinal and fecal DNA, respectively. Conventional PCR amplification of DNA extracted from the five ingredients and from dog food were all positive. The % of positive reactions is read on the left axis. qPCR estimates of fish and corn DNA concentration are indicated with dots. Concentrations are read on the right axis. Negative qPCR results are set equal 10^−7^ and vertically offset for clarity.

Similar analyses of food DNA digestion were performed with samples obtained through a clinical trial with dogs. As observed with the mouse samples, the degradation of DNA from these sources was apparent (Figure 1 and Figure 4). Corn was again partially resistant to digestion. Against expectations, chicken DNA was still present in the intestine, but not in the dogs’ feces (Figure 1). We again used qPCR to corroborate the interpretation of the gel images. Corn and fish were selected for this analysis because of their different rates of degradation observed with conventional PCR (Figure 1). Estimated concentrations of corn and fish broadly agree with the gel images. As expected from the higher sensitivity of PCR, fish DNA was detected at a low concentration of the dogs’ feces by qPCR.

## 4. Discussion

The goal of the research described here was to assess whether dietary records can reliably be inferred from the fecal food metagenome. The results obtained from the analysis of mouse and dog intestinal and fecal samples show evidence of different rates of DNA digestion, depending on the food source. Our hypothesis that, in contrast to animal DNA, plant DNA is protected from digestion by cell walls rich in insoluble fiber was only partially confirmed. Against expectations, chicken and to a lesser extent fish DNA persisted in the canine GI tract. As the mouse chow does not contain animal ingredients, it was not possible to assess the fate of DNA from animal sources in the mouse. Together, these observations demonstrate that a DNA-based inventory of food intake would only serve to confirm the consumption of a certain ingredient, but not to exclude the possibility that a food item was eaten, particularly in the case of food sourced from animals. Although not addressed in this study, the extent to which the consumption of highly processed food can impact the reliability of DNA-based dietary surveys is an additional concern. This potential drawback of DNA-based inventories was also acknowledged in a recent metagenomic survey of food DNA in the feces of participants in a controlled diet study [20]. The frequent consumption of such food, and the potential health consequences of diets rich in processed food [41], makes the inclusion of such foods in nutritional surveys particularly relevant. A more comprehensive analysis of diet was obtained with metagenome sequencing [20], as opposed to DNA markers, as performed here. Aside from the much higher cost of metagenome sequencing, this technique would not be able to overcome the potential bias resulting from the processing used in the production of industrialized multi-ingredient foods. Regardless of the sophistication of the DNA sequencing technique, dietary inventories based on DNA are unlikely to detect refined ingredients like sugar and perhaps oils and butter. On the other hand, processed food items like pasta and bread have been shown to contain DNA at concentrations potentially detectable with PCR [42,43,44]. These studies also show that DNA survives exposure to heat during cooking and baking. Contradicting these reports, our PCR assays did not detect DNA in autoclaved rodent chow, prompting us to perform all mouse experiments with non-autoclaved chow.

The use of antibiotics to perturb the mouse intestinal microbiota was intended to assess whether the intestinal microbiota impacts digestion bias. The breakdown of dietary DNA in the GI tract is likely to be accelerated by its release from plant and animal cells. Plant structural polysaccharides, such as pectin, inulin and cellulose are broken down by bacterial enzymes [45,46,47]. The importance of the bacterial metabolism in digestion [48] motivated us to compare the abundance of food DNA markers in antibiotic-treated and untreated mice. The quantification of corn DNA is consistent with this view. This ingredient was chosen for qPCR analysis because in our experiments it was particularly resistant to digestion. The unexpected lack of quantitative difference between the bacterial DNA concentration in antibiotic-treated and control mice does not exclude changes in the relative abundance of bacteria which are important for the digestion of dietary fiber. In the absence of sequence data, we do not have the ability to assess the impact of the antimicrobial treatment on the taxonomy of the microbiota populating various GI organs. The fact that mice were fed non-autoclaved chow could have increased the abundance of bacterial DNA or live bacteria in the GI tract and partially masked any antibiotic effect. The high concentration of 16S DNA we observed in chow is consistent with this possibility. If molecular methods were to be used to complement traditional food recalls and questionnaires, antibiotic treatment would have to be considered in the interpretation of DNA-based dietary information. Interventional studies targeting the microbiota would be needed to better understand the interaction between intestinal microbiota and digestion bias.

As all studies based on animal models, it is unknown to what extent the observations reported here apply to humans. While the data are broadly consistent with digestion bias, many variables remain to be explored with larger sample sizes for their effect on food DNA decay and persistence in the GI tract. A possible modification of the experimental design to increase the relevance of the study to human nutrition would be to replace rodent chow with a diet based on ingredients commonly used in human diet and processed according to common cooking procedures. In this way, the exact composition of the diet would be known, which would enable a more precise quantification of digestion bias. To study the connection between dysbiosis, as it may result from treatments with antimicrobials, and digestion bias, marker gene or shotgun metagenomics should be incorporated.

## 5. Conclusions

The quantification of DNA from several dietary ingredients in the mouse and dog intestine, and in the feces of these animals are indicative of DNA digestion bias. These observations lead us to postulate that DNA-based dietary inventories are unlikely to replace traditional questionnaires. Further limiting the scope of fecal DNA analysis is the impact of dysbiosis on DNA degradation in the GI tract and the unknown fate of food DNA during industrialized processing.

## Figures and Tables

**Table 1:** Positive controls, PCR primers for detecting main food ingredients and BLAST search results.

Ingredient ^1^	Primer Forward (5′→3′)	Primer Reverse (5′→3′)	Amplicon Top BLAST Hit	Reference
corn	atttgatcattatatacatttttgagat	tccttccttttttagagtattcc	*Zea mays* chloroplast	[35]
wheat	gaggggttttataccttatac	ggggatagagggacttgaac	*Triticum* spp. ^2^ chloroplast	[36]
soya	aataatagaatccttccgtc	ggggatagagggacttgaac	*Glycine soya* chloroplast	[36]
salmon	taagagggcggtaaaactc ^3^	gtggggtatctaatcccag ^3^	*Brevoortia tyrannus* ^4^ 12S rRNA	[37]
chicken	gggacaccctcccccttaatgaca	ggagggctggaagaaggagtg	*Gallus gallus* mitochondrion	[38]
yeast ^5^	agcatgagagcttttactg	tccagttacgaaaattct	*Saccharomyces cerevisiae* ITS	[39]

^1^ used to test primer specificity; ^2^ multiple wheat species; ^3^ generic fish primers; ^4^ Atlantic menhaden (order Clupeiformes); ^5^ not present in Purina ProPlan.

## Data Availability

The original contributions presented in this study are included in the article/Supplementary Material. Further inquiries can be directed to the corresponding author.

## Supplementary Materials

The following supporting information can be downloaded at https://www.mdpi.com/article/10.3390/nu17243865/s1, Table S1: PCR conditions for detecting food DNA; Figure S1: Wheat versus soya cross-specificity test; Figure S2: Replication of antibiotic effect on food DNA degradation in the mouse intestine; Figure S3: Increasing concentration of bacterial DNA in the GI tract of antibiotic treated and untreated mice.
