# Supplementary material for: Decay of Food DNA in the Gastrointestinal Tract: Implications for Molecular Dietary Records
Source: Nutrients. 2025 Dec 11;17(24):3865. doi: 10.3390/nu17243865 (PMC12735545; doi:10.3390/nu17243865)
Supplement: Supplementary file 1 [file nutrients-17-03865-s001.zip › nutrients-4017507-supplementary.pdf]

Table S1. PCR conditions for detecting food DNA

Ingredient	Dog	Mouse
wheat	95 °C for 5 m<br>38 cycles of 30 s @ 95 °C,<br>60 s @ 57 °C, 45 s @ 72 °C<br>72 °C for 5 m	98 °C for 30 s<br>33 cycles of 10 s @ 98 °C,<br>20 s @ 58 °C, 30 s @ 72 °C<br>72 °C for 3 m
soya	95 °C for 5 m<br>35 cycles of 30 s @ 95 °C,<br>30 s @ 57 °C, 30 s @ 72 °C<br>72 °C for 5 m	98°C for 30 s<br>33 cycles of 10 s @ 98 °C,<br>20 s @ 58 °C, 30 s @ 72 °C<br>72 °C for 3 m
corn	95 °C for 5 m<br>40 cycles of 30 s @ 94 °C,<br>30 s @ 55 °C, 30 s @ 72 °C<br>72 °C for 5 m	95° C for 5 m<br>38 cycles of 5 s @ 94°C,<br>30 s @ 57 °C, 10 s @ 72 °C<br>72 °C for 5 m
chicken	95 °C for 5 m<br>38 cycles of 30 s @ 94 °C,<br>30 s @ 58 °C, 30 s @ 72 °C<br>72 °C for 5 m	
fish	95 °C for 5 m<br>35 cycles of 30 s @ 94 °C,<br>30 s @ 54 °C, 30 s @ 72 °C<br>72 °C for 5 m	
yeast		95° C for 2 m<br>30 cycles of 30 s @ 95 °C,<br>30 s @ 57 °C, 30 s @ 72 °C<br>72 °C for 5 m

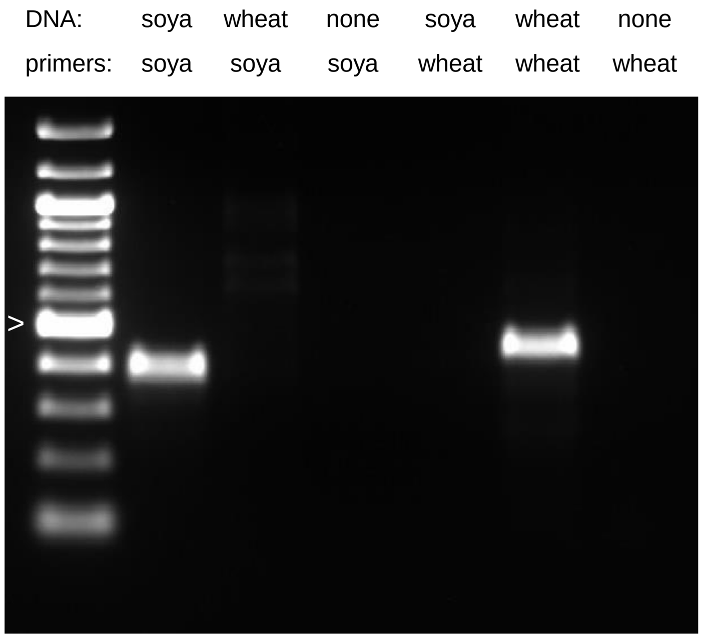

Figure S1. Wheat versus soya cross-specificity test.

PCR primer pairs specific for wheat and soya DNA (Table 1) were checked against matched and unmatched DNA.

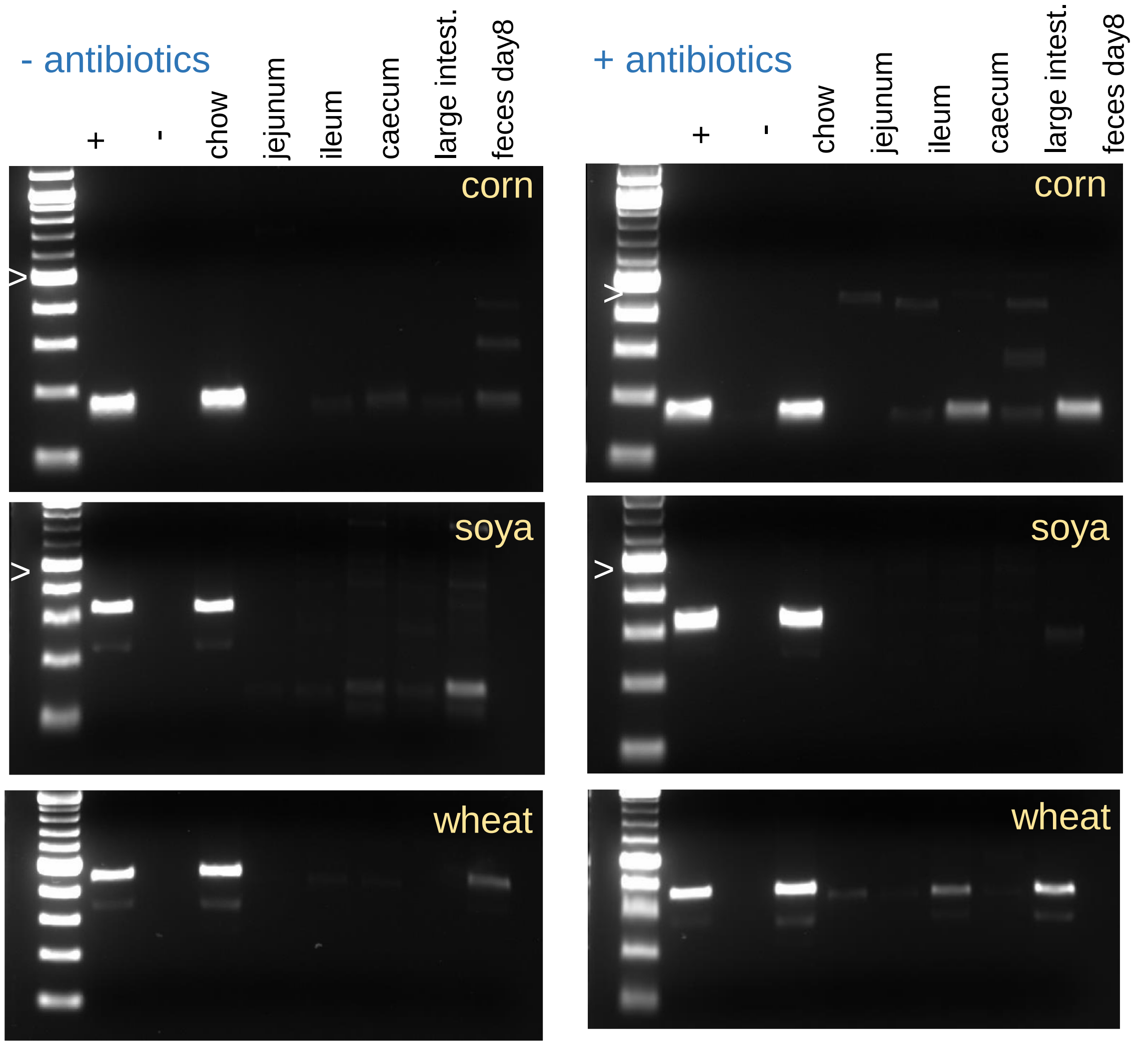

Figure S2. Replication of antibiotic effect on food DNA degradation in the mouse intestine.

Food DNA decay in a second pair of mice was analyzed to assess the reproducibility of the observations shown in Figure 2.

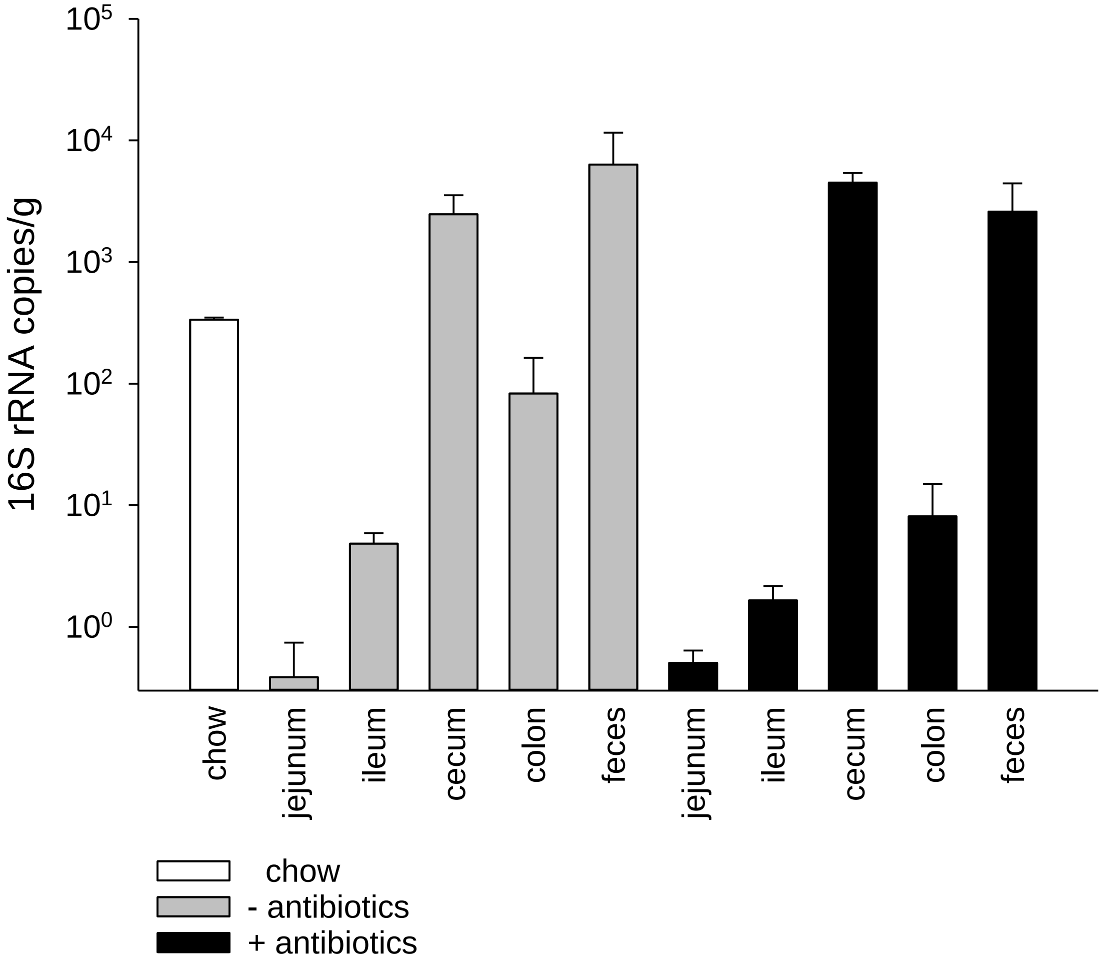

Figure S3. Increasing concentration of bacterial DNA in the GI tract of antibiotic treated and untreated mice.

Bacterial DNA concentration was estimated with qPCR using generic primers flanking the V4 variable region of the 16S rRNA gene. Each bar represents the mean of 2-7 concentration values inferred from qPCR crossing points. Error bars indicate SD.
